# Bg10: A Novel Metagenomics Alcohol-Tolerant and Glucose-Stimulated GH1 ß-Glucosidase Suitable for Lactose-Free Milk Preparation

**DOI:** 10.1371/journal.pone.0167932

**Published:** 2016-12-21

**Authors:** Elisângela Soares Gomes-Pepe, Elwi Guillermo Machado Sierra, Mariana Rangel Pereira, Tereza Cristina Luque Castellane, Eliana Gertrudes de Macedo Lemos

**Affiliations:** 1 Department of Technology, São Paulo State University (Unesp), School of Agricultural and Veterinarian Sciences, Jaboticabal. Via de Acesso Prof. Paulo Donato Castellane S/N, km 5, CEP, Jaboticabal, São Paulo State, Brazil; 2 Molecular Biology Laboratory; Institute for Research in Bioenergy (IPBEN), UNESP–Jaboticabal, SP, Brazil; 3 Agricultural Microbiology postgraduate program of UNESP, Jaboticabal, São Paulo State, Brazil; Weizmann Institute of Science, ISRAEL

## Abstract

New ß-glucosidases with product (glucose) or ethanol tolerances are greatly desired to make industrial processes more marketable and efficient. Therefore, this report describes the *in silico*/*vitro* characterization of Bg10, a metagenomically derived homodimeric ß-glucosidase that exhibited a V_max_ of 10.81 ± 0.43 μM min^-1^, K_cat_ of 175.1± 6.91 min^-1^, and K_*m*_ of 0.49 ± 0.12 mM at a neutral pH and 37°C when *p*NP-ß-D-glucopyranoside was used as the substrate, and the enzyme retained greater than 80% activity within the respective pH and temperature ranges of 6.5 to 8.0 and 35 to 40°C. The enzyme was stimulated by its product, glucose; consequently, the Bg10 activity against 50 and 100 mM of glucose were increased by 36.8% and 22%, respectively, while half of the activity was retained at 350 mM. Moreover, the Bg10 was able to hydrolyse 55% (milk sample) and 100% (purified sugar) of the lactose at low (6°C) and optimum (37°C) temperatures, respectively, suggesting the possibility of further optimization of the reaction for lactose-free dairy production. In addition, the enzyme was able to fully hydrolyse 40 mM of cellobiose at one hour and was tolerant to ethanol up to concentrations of 500 mM (86% of activity), while a 1 M concentration still resulted in 41% residual activity, which could be interesting for biofuel production.

## Introduction

β-glucosidases (EC 3.2.1.21) are enzymes that hydrolyse terminal, non-reducing, β-D-glucosyl residues with the release of glucose and shorter/de-branched oligosaccharide or aglycone compounds [1,2]. According to the Carbohydrate-Active enZYmes database (CAZy; http://www.cazy.org/), which classifies glycosyl hydrolases (GHs) based on sequence similarity, β-glucosidases can belong to the families GH1 and GH3. As determined experimentally for the enzyme GH1 Bgl3 [3], the GH1 catalytic site is formed by the two glutamate residues that are the most conserved of all known β-glucosidases belonging to this family. The first glutamic acid residue is related to the overall acid/base interaction with the β-(1,4) glycosidic bonds, whereas the second glutamate residue is involved in the catalytic nucleophilic action of the retention mechanisms. In contrast, β-glucosidase from the GH3 family utilizes one aspartate for a nucleophilic attack and one glutamate as a proton donor [3,4].

The various biotechnological applications of β-glucosidases include their increasing use in the development of second-generation biofuels and food industrial processes [5–12]. Moreover, ß-glucosidases perform transglycosylations in addition to their hydrolysis activities, making them suitable for applications in the synthesis of glycosylated compounds such as ginsenoside and other stereo- and region-specific glycosides, which are, in turn, potentially useful as nutraceutical or pharmaceutical compounds [2,13,14].

Biofuel development is encouraging attempts to increase production without expanding agricultural boundaries and causing damage to food crops [6–9]; these attempts include the generation of energy from industrial waste, usually lignocellulosic biomass. However, this second-generation technology is advancing at only a moderate speed due to the high costs associated with processes involving enzymes [10,15,16]. In general, the degradation of lignocellulosic biomass involves interactions among endocellulases, exocellulases, and ß-glucosidases. The last group of enzymes catalyses the final step in the release of glucose from cellobiosidic molecules and increases the efficiencies of endocellulases and exocellulases, which are often product-inhibited by these compounds [11].

In the food and beverage industry, ß-glucosidase triggers the release of flavouring agents due its hydrolytic activity on glycosidic conjugates. This process is largely applied in wine and juice production and involves the liberation of flavouring compounds from certain precursors, such as glycoside monoterpenes (linalool, geraniol, and nerol) [12,17,18]. However, the application of ß-glucosidases could be greatly extended by using many enzyme activities to modify sugars in food products, such as an enzyme’s lactase activity. The lactase activity is important to the food market for products with low lactose content, as half of the world’s population suffers from malabsorption or lactose intolerance [19–21]. Actually, despite the fact that some ß-glucosidases are completely suitable for this process, low-lactose or lactose-free milk and their derivatives are usually prepared using ß-galactosidases, which are generally inhibited during the reaction as the glucose concentration increases [22].

Independent of the application process and regarding product effects on enzyme reactions, one key characteristic of β-glucosidases is their relationship to the product–glucose. The great majority of β-glucosidases are inhibited by this product [2,11,23–25], but some β-glucosidases are tolerant to [2,26,27] or even stimulated by [28,29] glucose. Understandably, enzymes with glucose tolerance are greatly desirable in industrial processes, as this characteristic affects substrate conversion [2,30]. The mechanisms by which the product affects the enzyme were recently attributed to the accessibility of the active site to glucose and the energy that allows this molecule to bind to different sites in the protein [2,31]. Thus, in the first situation [31], the more neutral or even electropositive formal charge that maintains residues present in some β-glucosidases could make access by glucose difficult, leading to glucose tolerance. Moreover, all GH3 β-glucosidases are glucose sensitive and possess shallow pockets, whereas glucose-tolerant GH1 β-glucosidases possess deep pockets. This evidence suggests that pocket depth might also interfere with glucose access to the active site [31]. For the second theory [2], these features may not only affect glucose tolerance but may also promote more affinity of the glucose for certain sites within the protein with different binding energies. Therefore, based on mutant constructions, kinetic proprieties, and computational assays of docking and homology-modelling protein predictions, this theory highlights strong evidence that the glucose bound to the active site could lead to inhibition, whereas glucose with an affinity for the protein pocket entrance or middle could lead to tolerance or a stimulation effect, respectively [2].

Despite the many approaches to large-scale sequencing and metagenomics leading to the generation of a large and growing databases of protein sequences [32–38], the fraction of enzymes that have been experimentally characterized is very small [39,40]. For instance, the data on glycosyl hydrolases (GHs), which are classified based on sequence similarity, deposited in CAZy consist of 194,596 entries, distributed across 127 families, and 2,314 sequences that are non-classified. From this total, only 3.5% (6,891) of GHs have been experimentally characterized *in vitro*, and the structures of just 0.5% (938) have been experimentally elucidated.

Taking into account the relevance of metagenomic approaches for identifying new enzymes and the importance of ß-glucosidases to the industrial process, this report describes the *in silico* and *in vitro* characterization of Bg10, a metagenomically derived ß-glucosidase that was obtained using *in silico* strategies for homology-based annotational inference [41]. The open reading frame (ORF) for this enzyme (*ORF10* from the B5p37metaSE clone) was identified from a DNA library of soil from beneath *Eucalyptus* sp. forest litter [41,42] and shared 81% identity with *Streptomyces hygroscopicus* (gb|CP003720.1| NCBI Data Bank entry). The construction of protein structure models based on homology (template 1GNX, with 70% identity) and the identification of conserved catalytic residues combined with heterologous expression and a kinetic study of Bg10 revealed that this enzyme is an alcohol-tolerant and glucose-stimulated ß-glucosidase with high lactase activity that has a great deal of potential as a biotechnological product in the dairy industry [22].

## Material and Methods

### Sample origin and three-dimensional protein structure prediction

The studied material was retrieved from a metagenomic library developed from DNA obtained from soil under eucalyptus litter [41]. Following sequencing of the cosmid clone (Sanger method, shotgun sub-library strategy) and the assembly of reads obtained from a FASTA sequence [43], ORFs were identified using the ORF Finder program and were subjected to manual screening based on their greatest similarity to known sequences. The amino acid sequence deduced for the gene of interest (ORF10, Bg10 ß-glucosidase clone B5P37metaSE, GenBank accession KX364386) was uploaded to the ‘Swiss-Model’ server [44] to generate a three-dimensional model of the protein using the *‘Swiss-Model Automatic Modelling Mode’*. In addition, the ‘*ProtParam*’ tool [45] was used to predict the molecular mass and molar extinction coefficient for the protein in its monomeric state.

### Phylogenetic analysis

One phylogenetic tree was built to analyse the phylogenetic relationships of Bg10. For this process, 103 sequences for GH1 (E.C. number 3.2.1.21), corresponding to all bacterial-characterized β-glucosidases according to the CAZy database, were retrieved from NCBI (https://www.ncbi.nlm.nih.gov/sites/batchentrez). The sequences were aligned with Bg10 on the Mobyle Pasteur Web server (http://mobyle.pasteur.fr/cgi-bin/portal.py#jobs::clustalw-multialign.S59285820297003) using the ClustalW program (2.0.12 version; multialign with open gap 15, extend 0.3 and matrix Gonnet 250). After retrieving the alignment, the file was submitted to the package Phangorn from the R software [46] to investigate the best matrix to analyse the set of data. Once predicted, the best matrix for amino acid substitution (LG G+I) was used to construct the tree using the “Mr. Bayes” software on the Cipres Web server (https://www.phylo.org/portal2/tools.action) to simulate a suitable evolutionary model. The phylogenetic construction method used was the Bayesian model with four chains to run (*nchains*), two billion cycles for the MCMC algorithm runs (*Ngen*) and two independent analyses started simultaneously (*nruns)*. The value for Ngen was adjusted to obtain one average standard deviation of split frequencies up to 0.015 [47].

### Heterologous expression and purification of the recombinant protein

For *ORF10* gene cloning, the following *forward* (F) and *reverse (R)* oligonucleotide primers were synthesised: F5′-TGG*GAATTC*AGTCCCCGCATGACAGC-3′ and R5′-CAC*AAGCTT*CGTTCCTTCAGGACGTC-3′, containing restriction sites for EcoRI and Hind III, respectively. The resulting amplicons were digested with the appropriate restriction enzymes and purified (Wizard® SV Gel and PCR Clean-Up System; Promega, Madison, WI, USA). The fragments were then cloned into the pET28a vector (Novagen, Madison, WI, USA) and transformed into *Escherichia coli* BL21 DE3 competent cells. The recombinant protein was purified by affinity chromatography using nickel resin Ni-NTA (Qiagen, Venlo, Netherlands) in 9-cm Poly-Prep chromatography columns (Bio-Rad, Hercules, CA, USA) following the manufacturer’s instructions for extraction and purification. Molecular exclusion chromatography (Äkta pure; column HiLoad 16/60 Superdex 200, GE Healthcare) was performed to estimate the molecular size of ß-glucosidase Bg10. Fractions of 2 mL were collected at a flow rate of 0.8 mL min^-1^, and samples corresponding to peaks in the UV chromatogram light (280 nm) were analysed by sodium dodecyl sulfate polyacrylamide gel electrophoresis (SDS-PAGE). The elution profile of the band of interest was compared to the profile of the marker standards (GE).

The total protein concentration was assessed using the Bradford method (1976) based on a standard curve prepared using bovine serum albumin (BSA). The concentration of pure protein was calculated from the absorption measured at 280 nm using a NanoDrop 1000 spectrophotometer (Thermo Scientific, Waltham, MA, USA) based on values of the molar extinction coefficient and the molecular mass of the metagenomic protein calculated using the ProtParam program. The extracted and purified samples were analysed using denaturing polyacrylamide gel electrophoresis [48]. Protein separation was achieved at 125 V and 25 mA. The samples were stained using Coomassie blue solution (0.2% Coomassie blue, 40% methanol, 10% acetic acid). For enzyme activity detection by zymogram, aliquots of the purified protein were also electrophoresed under non-denaturing conditions (in the absence of SDS and ß-mercaptoethanol; without heating) in an ice bath. Next, a portion of the gel containing an aliquot of the enzyme was immersed in sodium phosphate buffer (20 mM, pH 7) with the addition of 5 mM of *p*NP-ß-D-glucopyranoside (*p*NP-βG; (Sigma, St. Louis, MO, USA); the mixture was then incubated at 37°C for 20 min to reveal the catalytic activity (zymogram). Another portion of the gel containing BSA and an aliquot of the sample was stained using Coomassie blue.

### Relative enzyme activity assay using chromogenic substrates

To determine the enzyme activity on chromogenic substrates, the absorbance was measured at 405 nm to estimate the release of p-nitrophenolate ions (*p*NP, ε405 = 0.0187 μM cm^-1^, pH 10) following a reaction [40]. The initial reaction (100 μL) comprised 82 μL of 20 mM sodium phosphate buffer, pH 7, including 1 mM substrate (final concentration) and 18 μL of Bg10 at a concentration of 0.02 mg mL^-1^. The addition of the enzyme to the reaction mix was timed at 1-min intervals. The reactions were incubated in a thermal cycler at 37°C or at the temperatures specified in the description of the respective experiments. Following incubation under the relevant assay conditions, 30 μL aliquots were added to 150 μL of sodium carbonate buffer (500 mM, pH 10) in 96-well, flat-bottomed, tissue culture plates (JetBiofil, Guangzhou, China) and homogenised by pipetting with a semi-automatic multichannel pipette. The released *p*NP was immediately measured at 405 nm using a SpectraMax^®^ M2^e^ spectrophotometer (Molecular Devices, Sunnyvale, CA, USA). All assays were performed in triplicate, and the following controls were used: reactions without enzyme and reactions without the assayed compound. The enzyme activity was expressed in percentages relative to the highest value or to the specified standard for each essay.

### Optimal temperature and pH and determination of kinetic parameters

To determine the optimal reaction conditions for the purified Bg10, ß-glycolytic activity assays were performed in triplicate at a pH ranging from 3.5 to 10.5 and temperatures ranging from 4°C to 50°C. The relative activity was expressed as the percentage of the activity found under the optimal conditions for each assay.

For the kinetic assay, the activity of purified Bg10 was determined in triplicate for various concentrations of *p*NP-ßG (0.1–10 mM) under optimal determined conditions, and the activity was measured from 0–60 min at 10-min intervals. The initial velocity (V_0_) of the *p*NP formation was calculated using the linear regression equation obtained for the plot of the product formed as a function of time; thus, V_0_ is equal to the slope of the line obtained for the linear interval corresponding to each substrate concentration. Kinetic parameters *K*_m_ (Michaelis-Menten constant) and *V*_max_ (maximum reaction velocity) for the substrate *p*NP-ßG were calculated by a nonlinear regression of the Michaelis-Menten equation using GraphPad PRISM version 5.0 (GraphPad Software, La Jolla, CA, USA).

### Effect of salts and other reagents on enzyme activity

The effect of salts (and corresponding ions) on the Bg10 activity was assessed by pre-incubating 18 μL of enzyme, which was conveniently diluted with 82 μL of sodium phosphate buffer (20 mM, pH 7.0), in PCR microplates at 37°C for 10 min; the solutions contained the appropriate amounts of salts to achieve final concentrations of 0–5 mM. After the pre-incubation period, 20 μL of the substrate (10 mM pNP-ßG) were added to each well and homogenised by successive pipetting with a multichannel pipette. The samples were incubated for a further 30 min at 37°C; then, 30-μL aliquots were added to 150 μL of sodium carbonate buffer (500 mM, pH 10) and homogenised by pipetting, realising *p*NP estimates as previously described (section 2.3). In a similar way, the enzyme activity was assayed for the influence of alcohols, glucose, SDS and ß-mercaptoethanol.

### Bg10 activity for natural substrates and ‘zero lactose’ milk assay

The hydrolysis product of the metagenomic β-glucosidase for natural substrates was identified by high-performance liquid chromatography (HPLC). Cellulose (20 mg/mL), cellobiose and lactose (40 mM) were used as substrates for the characterization of the β-glucosidase. The enzymatic reaction mixture contained each substrate, 0.05 M sodium phosphate buffer at pH 7.0, and approximately 100 μg purified recombinant protein. The reaction was conducted in a total volume of 0.5 mL at 37°C for 1 hour, under agitation (300 rpm min^-1^). On termination of the reaction with 0.5 mL 1 M Na_2_CO_3_, the residual protein was removed by centrifugation through a membrane (Vivaspin 500, Vivascience, USA). The filtered reaction sample was separated on a Supelco Analytical Column (Sigma, #117815.06), eluted with double-distilled H_2_O and acetonitrile (25:75, v/v) in the mobile phase at a flow rate of 1.0 mL min^-1^ and detected using an HPLC system equipped with a UV–vis spectrophotometer (Shimadzu, model SPD-M10A). The detection wavelength was 245 nm, and the monosaccharide glucose was used as the standard at 50 and 100 μg mL^−1^.

Bg10 was also assayed as the active lactase enzyme for the production of milk with reduced lactose or without lactose (‘zero lactose’ assay). For this, 1 mL-samples of milk (Jussara, Patrocínio Paulista, SP, Brazil) were incubated at 25 or 6°C for 1 or 15 hours, respectively, with 100 μg of purified Bg10. The sugar contents from the treated samples and controls (without enzyme and commercial ‘zero lactose’ milk: Jussara, Patrocínio Paulista, SP, Brazil) were extracted according to the milk sugar extraction protocol optimised by Acquaro Jr. and co-workers [49], which consisted of the addition of 5 mL of absolute ethanol for each mL of milk, followed by centrifugation at 12,857 x g for 37 min at 10°C. Thus, the supernatants were collected and submitted to complete evaporation by vacuum concentration (Concentrator Plus, Eppendorf, Hamburg, Germany). Subsequently, the samples were re-suspended in 500 μL of double-distilled H_2_O ultra-purified water, filtered through 0.22 μm nitrocellulose filter paper (Millipore) and were analysed for natural substances by HPLC as described above.

All data obtained were analysed using the R software. ANOVA and Tukey’s test at 5% probability were used to compare the treatment methods.

## Results and Discussion

### Eucalyptus soil metagenomically derived Bg10: phylogenetic relationship, protein structure and function predictions from homology

The Bg10 ß-glucosidase was derived from the B5p37metaSE cosmid clone that was retrieved by a sequence-driven prospective approach for antibiotic gene clusters from a DNA library of soil from beneath *Eucalyptus* sp. forest litter [41]. The *Eucalyptus* sp. forest that was the source of the DNA library was planted on the UNESP campus in the city of Jaboticabal, state of Sao Paulo, Brazil, in February 1969 and had not been submitted to any crop management for 40 years before the study was conducted [41]. This same area was submitted to previous study for verification of the biodiversity of the microbial communities by 16S rDNA sequencing. The results indicated that this soil predominantly harboured sequences of Actinobacteria, Firmicutes, and Verrucomicrobia, whereas 72% of the analysed sequences were novel, suggesting an important source of new enzymes [42].

The 23-kb-length cosmid DNA insert B5p37metaSE was submitted to sequence comparison with GenBank sequences by use of the BLAST tool provided by the National Center for Biotechnology Information (NCBI: http://www.ncbi.nlm.nih.gov), and the results demonstrated that only 38% (query coverage) of this sequence was similar to other sequences deposited in the NCBI database but shared 78% identity with *Kitasatospora setae* KM-6054 (AP010968.1 NCBI code), an Actinobacteria from *Streptomycetaceae* family. The gene annotation for B5p37metaSE showed that the metagenomic DNA fragment harboured not only one sequence for an aromatic polyketide antibiotic cluster but also sequences related to carbohydrate degradation: one ß-glucosidase (ORF10, corresponding to Bg10 enzyme, target of this study), one chitinase (ORF11), and one laccase (ORF12).

According to S1 Table, which shows the amino acid sequences more similar to Bg10 from different databases, Bg10 had more similarity (80–88%) with the glycosyl hydrolases from *Streptomyces sp*., and this similarity was observed with both ß-galactosidase and ß-glucosidase. However, this classification could be wrong after these closest sequences corresponding to not-yet-characterized enzymes have been analysed. Moreover, on the basis of amino acid sequence similarities, these two enzymes are grouped in the same family with glycosyl hydrolase 1 (GH1). Although enzymes belonging to the same family appear to share common mechanistic features, subtle differences in the fine-tuning of the enzyme activity are expected, and the same enzyme might perform two or more actions in addition to displaying diverse substrate preferences. Thus, identification of the particular molecular mechanisms for new enzymes requires detailed structural, kinetic, and mechanistic studies of a larger number of glycosidases [3].

**Fig 1:**
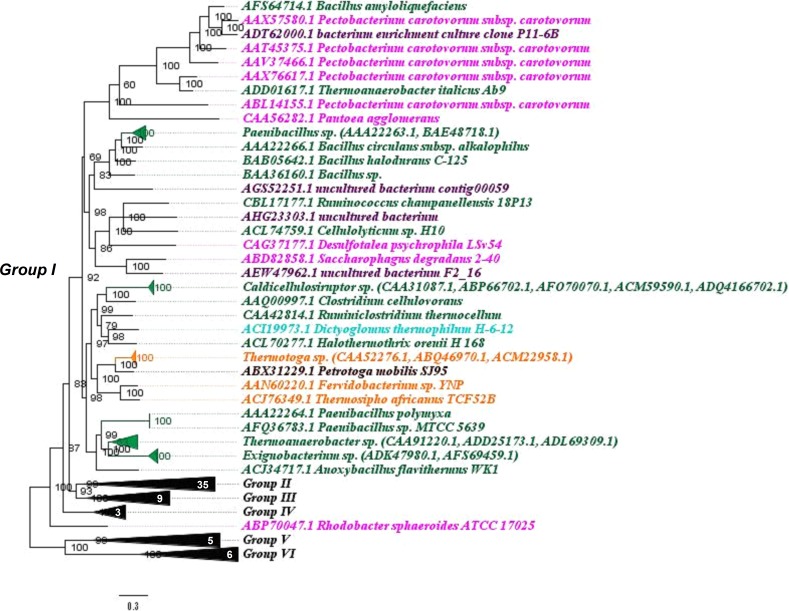
“Group I view”: Phylogenetic relationship between Bg10 and other previously characterized GH1 β-glucosidases. The tree was constructed using the Bayesian model with algorithmic tests to determine the better amino acid substitution matrix (Phangorn at “R” software) and phylogenetic model (Mr Bayes software) using two billion generations (Ngen) to find the better method. The scale bar indicates the number of amino acid substitutions per site. In this view, the focus is on the related group I. The sequence for Bg10 fell in group II. Observation: for a complete tree view, see S1 Fig; for visualization of other groups, see also Figs 2 to 4. Colour code: Firmicutes, dark green; Proteobacteria, pink; Thermotogales, orange; Dictyglomales, light blue; Petrotogales, brow; unculturable, purple. Numbers in white specify the amount of sequences in each collapsed branch.

**Fig 2:**
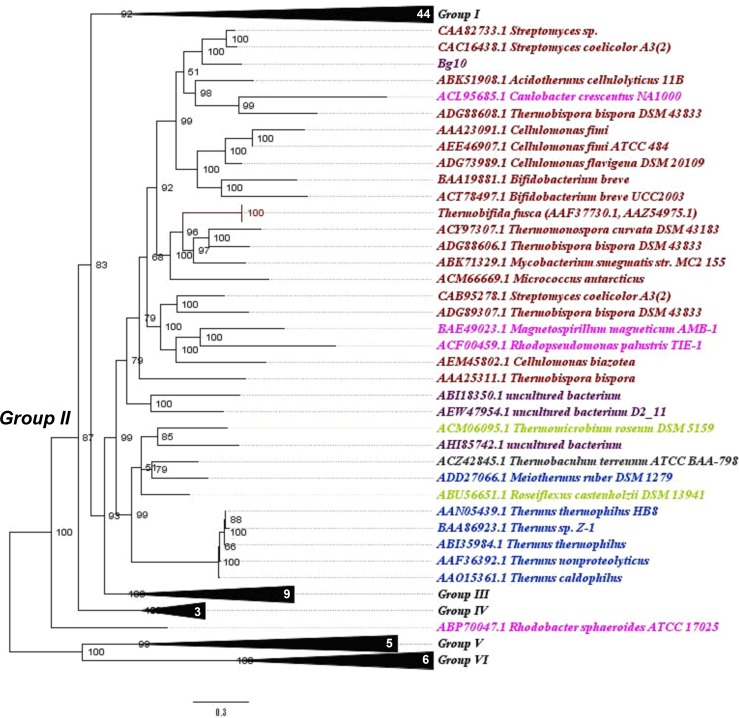
“Group II view”: Phylogenetic relationship between Bg10 and other previously characterized GH1 β-glucosidases. The tree was constructed using the Bayesian model with algorithmic tests to determine the better amino acid substitution matrix (Phangorn at “R” software) and phylogenetic model (Mr Bayes software) using two billion generations (Ngen) to find the better method. The scale bar indicates the number of amino acid substitutions per site. In this view, the focus is on the related group II, including the Bg10 sequence. Observation: for a complete tree view, see S1 Fig; for visualization of other groups, see also Figs 1, 3 and 4. Colour code: Actinobacteria, red; Proteobacteria, pink; Thermales, dark blue; “Novel green non sulphur bacteria” (NGS), light green; Thermobaculum, grey; unculturable, purple. Numbers in white specify the amount of sequences in each collapsed branch.

**Fig 3:**
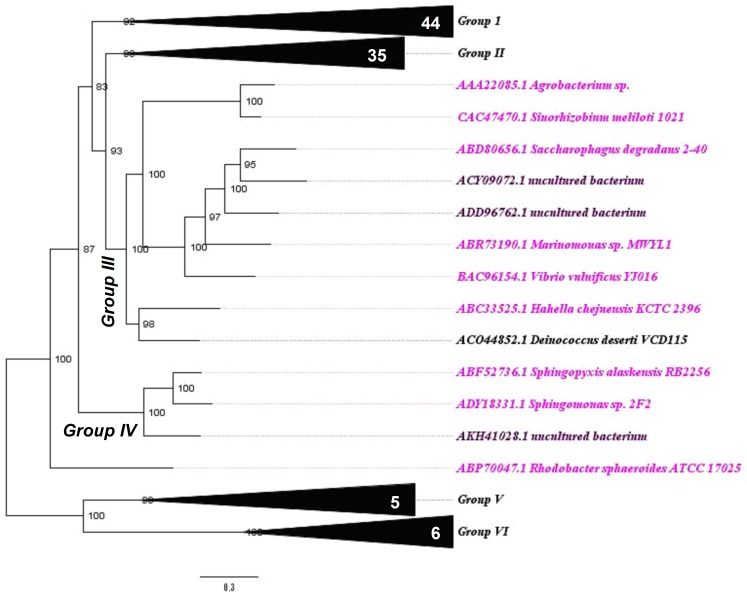
“Group III and IV view”: Phylogenetic relationship between Bg10 and other previously characterized GH1 β-glucosidases. The tree was constructed using the Bayesian model with algorithmic tests to determine the better amino acid substitution matrix (Phangorn at “R” software) and phylogenetic model (Mr Bayes software) using two billion generations (Ngen) to find the better method. The scale bar indicates the number of amino acid substitutions per site. In this view, the focus is on the related groups III and IV; for the complete tree see S1 Fig and Figs 1, 2 and 4. The sequence for Bg10 fell in group II. Colour code: Proteobacteria, pink; Deinococcales, black; unculturable, purple. Numbers in white specify the amount of sequences in each collapsed branch.

**Fig 4:**
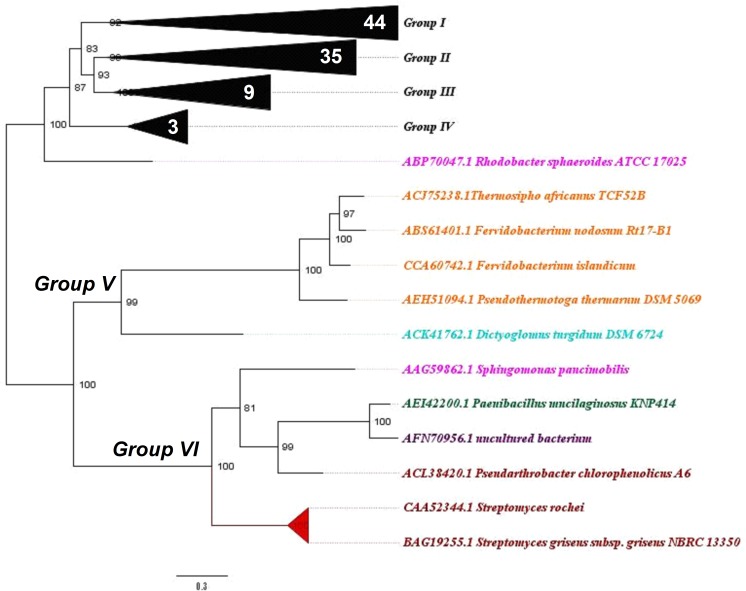
“Group V and VI view”: Phylogenetic relationship between Bg10 and other previously characterized GH1 β-glucosidases. The tree was constructed using the Bayesian model with algorithmic tests to determine the better amino acid substitution matrix (Phangorn at “R” software) and phylogenetic model (Mr Bayes software) using two billion generations (Ngen) to find the better method. The scale bar indicates the number of amino acid substitutions per site. In this view, the focus is on the related groups V and VI; for the complete tree see S1 Fig and Figs 1, 2 and 4. The sequence for Bg10 fell in group II. Colour code: Firmicutes, dark green; Actinobacteria, red; Proteobacteria, pink; Thermotogales, orange; Dictyglomales, light blue; unculturable, purple. Numbers in white specify the amount of sequences in each collapsed branch.

To verify the phylogenetic relationships between Bg10 and other previously characterized β-glucosidases available in the CAZy database, a Bayesian phylogenetic tree was built as described in the materials and methods section (2.2). A well-supported tree was generated, with good results for branch support and standard average. The complete version of the tree is shown in S1 Fig. For didactic purposes, this tree is also presented in partial views, focusing on six more segregated groups, displayed in Figs 1–4.

Among the characterized GH1 β-glucosidases, the most abundant enzymes were from the Firmicutes, Actinobacteria and Proteobacteria phyla, which are the sources of 25, 22 and 20% of the analysed sequences, respectively. Except for one sequence (AEI42200.1 NCBI code), all of the enzymes from the Firmicutes phylum were in group I, in addition to the sequences from Proteobacteria and Thermotogales. The majority of sequences from Actinobacteria were found in group II, while the Proteobacteria were found in every group except group V. The sequence for Bg10 fell in group II and was most closely related to two sequences from *Streptomyces* genera (69% identity), corresponding to the β-glucosidases Bgl3 and SC7558. Information of these enzymes and other enzymes closely related to Bg10 is shown in Table 1. The data shown for Bg10 were obtained from the experiments further described throughout this work. Even though the sequences represents related enzymes, they not shared the same properties, neither the similarity between them was too high (46–69%). These comparisons indicates that Bg10 own a combination of some proprieties not previously identified in others characterized enzymes. This set of characteristics may be valuable to explore new possibilities at industrial enzyme application. Also, this work could be extremely useful for functional predictions for not yet characterized enzymes closest to Bg10, as the NCBI entries WP_033177502.1, WP_031523150.1 and WP_037907004.1 (80–88%) found in S1 Table.

**Table 1:** Comparison of the properties of Bg10 with the phylogenetically closest β-glucosidases.

Name	Source	pH/ °C	Size (KDa)	Highlight properties	% I(QC)*	Reference
Bg10	Metagenomic (KX364386.1)	7.0/37	116.6	Alcohol resistance; Glucose Stimulation/tolerance; lactase activity, high stability front many ions.	-	This work
Bgl3/ 1GNX	*Streptomyces sp*. QM-B814 (CAA82733.1)	6.5/50	52.6 (dimmer at crystal)	Alcohol resistance, glucose inhibition (K_i_ 65mM).	69(95)	[3,4,50,51]
SC7558	*S*. *coelicolor* A3(2) (CAC16438.1)	6.0/35	52.2	Better β-glucosidase from this organism. The protein was stable over a broad range of pH (3.0–10.0).	69(95)	[52]
*Cbg*	*Cellulomonas fimi (*AAA23091.1)	NR	NR	Cellobiose and aryl-glucoside hydrolysing enzyme.	6t6(72)	[53,54]
I	*Bifidobacterium breve* (BAA19881.1)	5.5/45	48	Increasing activity by acclimation with cellobiose; inhibition with glucose.	51(93)	[55,56]
CldC	*B*. *breve UCC2003* (ACT78497.1)	7.0/37	52.1	Not capable of hydrolyzing sucrose and lactose; hydrolyzing cello: -biose, -triose, -tetraose and -pentaose.	46(94)	[57]

NR: not reported

* I: ident, QC: query coverage. Observation: Although they were designed as “characterized enzymes” by the database CAZy, it was not possible to find any reference directly linking enzyme studies to the sequences for accession numbers ACL95685.1, ADG73989, ADG88608.1, AEE46907.1, or ABK51908.1. Instead, they were only found in genome sequencing articles or by homologous enzyme predictions. For more information, see Fig 2.

Table 1 covers only bacterial sequences. For other sources of β-glucosidases, including those from fungi, plant and animals, there is one recent and good review which might be very useful [58]

**Fig 5:**
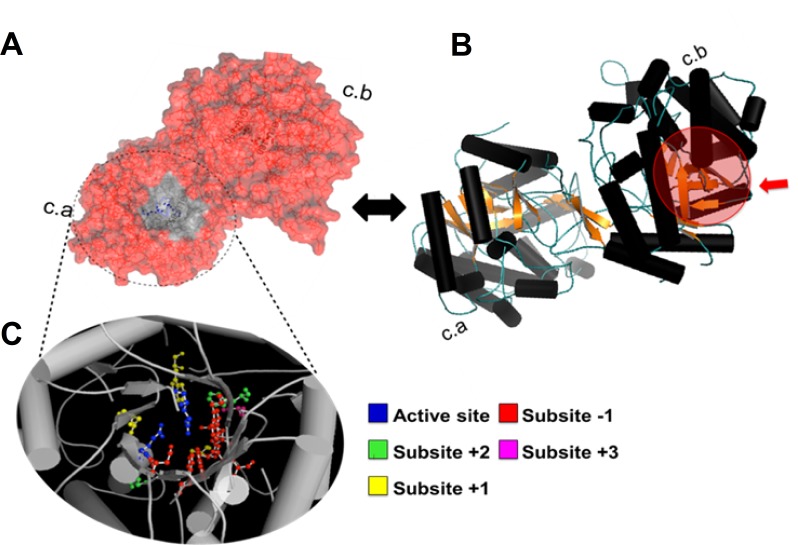
Sequence and structural features of metagenomic ß-glucosidase (Bg10). **A:** Sequence homology-based prediction of protein folding for the Bg10 homodimeric structure using the tool ‘Automated Mode Modelling’ from the Swiss Model Web-server, which was developed on the basis of a similar template, 1GNX (Bgl3 β-glucosidase) and represents the protein surface, showing one enzyme pocket entrance (circle). The two entrances from each protein chain cannot be visualized together because they are asymmetrically arranged, opening in opposite directions. **B:** The Bg10 predicted structure is represented by a cartoon view, where each protein chain (ca and cb) exhibits a TIM barrel (α/β) 8 super-secondary organizational motif, which is canonical for GH1 enzymes [39]. The chain b pocket entrance is indicated by the red sphere. **C:** Details of the functional pocket, showing the active site and residues from the -1 to +3 subsites, which were predicted on the basis of amino acid alignment with the experimentally characterized *Streptomyces sp*. Bgl3 β-glucosidase [34, 38].

For the sequences that already have had their protein fold solved experimentally, a higher hit was obtained for the ß-glucosidase Bgl3, corresponding to the structure for PDB protein 1GNX (69%). The good similarity result for that structure favoured a prediction of the protein structure for Bg10 by a homology-modelling approach (Fig 5A and 5B), using the Swiss-model server [44,59].

The predicted model exhibits a homodimeric form. The entrance for each pocket is oriented in an opposite direction from the other (Fig 5A), and each chain separately configures an entire activity pocket, so there are two activity sites per protein that seem to work independently. Despite these results, the protein`s oligomeric shape prediction by homology was accepted with reservation, as these features are very difficult to predict based only on amino acid sequence analysis, but further purification and chromatography results confirmed the dimeric form of Bg10 (section 3.2). Each chain formed a TIM barrel with an (β/α)8 tertiary structure (Fig 5B) that is shared by all known β-glucosidases [60]. The principal residues for the pocket cavity within the active site of Bg10 (Fig 5D) were predicted on the basis of sequence alignment with previously identified ß-glucosidases, which were studied of their kinetic, mutational, and catalytic properties [3,60]. The GH1 β-glucosidase active sites are composed of several subsites that are large enough to bind to a monosaccharide unit. The subsite that binds the monosaccharide of the substrate non-reducing end is denominated subsite -1 (or the glycone subsite), whereas the remaining part of the substrate is accommodated in the aglycone binding region, which may be formed by several subsites (such as +1, +2 and others). According to the proposed mechanism, the interaction of the enzyme with the glycosidic substrate is mediated by the side chain carboxylic acids of two glutamate residues that are present in the active site; that is, the acid/base catalyst, the catalytic nucleophile and the substrate cleavage occur in the point between the glycone (bound to subsite -1) and its aglycone/or other moieties binding region (bound to the others subsites). Later studies showed that these residues are extremely conserved among the ß-glucosidases that are members of the GH1 family [60].

### Purified recombinant Bg10 was active in the homodimeric form

**Fig 6:**
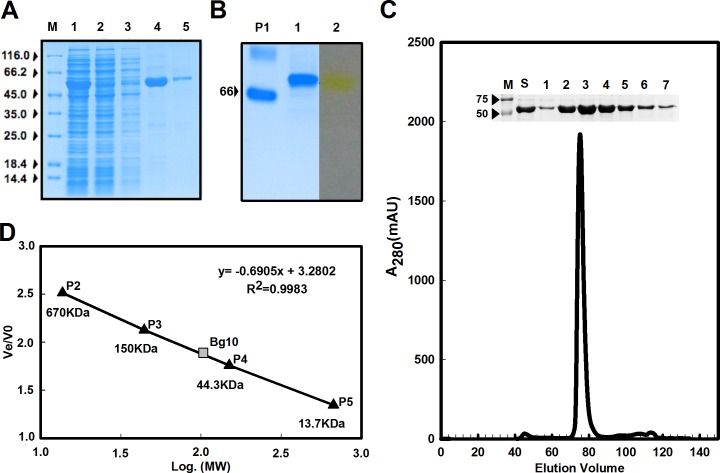
Extraction, purification and hydrolytic activity of recombinant Bg10 ß-glucosidase from *Escherichia coli* BL21(DE3) host cells carrying the pET28a-bg10 vector. **A:** Electrophoretic profile of Ni-NTA affinity chromatography purification fractions of Bg10 under denatured conditions in an SDS-PAGE gel (10% polyacrylamide). M: molecular weight standards (Thermo Scientific). Lane 1: soluble extract of the induced cells. Lane 2: flow-through fraction from affinity chromatography. Lanes 3 to 5: eluted fractions with 20, 500 and 100 mM imidazole, respectively. **B**. Zymogram of recombinant Bg10 under non-denaturing conditions. P1: Protein used as molecular weight standard, ‘Bovine Serum Albumin’ (BSA, Sigma, St. Louis, MO, USA). Lane 1: purified protein. Lane 2: ß-1-4-glycosidic activity of purified Bg10 band using *p*NP-ß-D-glucopyranoside as substrate (Sigma, St. Louis, MO, USA). **C:** Chromatographic profile of Bg10 by gel filtration using a Superdex 16.600.200 column (GE healthcare) at a flow rate of 0.5 mL/min. The internal image corresponds to the electrophoretic migration profile in an SDS-PAGE gel (10% polyacrylamide) for the analysed samples. M: molecular weight standards (Bio-Rad, Hercules, CA, USA). S: Sample of Bg10 before the gel filtration chromatography assay. Lanes 1 to 7: eluted fractions from gel filtration for the enzyme Bg10. **D:** Estimation of Bg10 molecular size by gel filtration based on the linear correlation of the relative migration patterns of Bg10 and protein standards versus their log molecular size values. P2-P5: Proteins used as molecular weight standards, corresponding to thyroglobulin bovine, ƴ-globulin, albumin, ribonuclease A and P-aminobenzoic acid, respectively (Protein Standard Mix 15–600 kDa, Sigma, St. Louis, MO, USA).

The Bg10 recombinant protein was obtained by heterologous expression in *Escherichia coli* BL21 (DE3) host cells carrying the pET28a-bg10 vector and was wholly purified by two chromatographic steps: Ni-NTA affinity chromatography and size exclusion chromatography (Fig 6A–6C). The Ni-NTA affinity chromatography resulted in one high-purity 500-mM sample from the imidazole elution fraction (Fig 6A), and as shown in the SDS-PAGE assay in a denatured state (Fig 2A and 2C), one chain of Bg10 displayed more the 50 kDa (~55 kDa). As shown with the zymogram electrophoretic gel in native condition (Fig 6B) for the desalted sample of Bg10 from the Ni-NTA affinity chromatography, only one band appeared in the gel without evidence of oligomeric polymorphisms, and this protein band had ß-glycosidic action towards the *p*NP-ß-D-glucopyranoside substrate. In the polishing step of the chromatography (Fig 6C), the enzyme was obtained in pure fractions that were pooled and used in subsequent enzymatic assays.

As expected from the results of the protein modelling prediction by homology (section 3.1), Bg10 might have one dimeric form. Because the deduced amino acid sequence that corresponded to only one protein copy was analysed using the ProtParam tool (http://web.expasy.org/protparam/) and the molecular mass of the polypeptide was estimated to be 58.33 kDa, the expected homodimeric form could correspond to the molecular size of 116.66 kDa. This prediction was confirmed with the linear regression for the migration of the Bg10 and protein standards in the size exclusion chromatography (Fig 6D), which allowed estimation of the molecular weight for an enzyme corresponding to 103.12 kDa; thus, the value of 116.66 kDa was adopted for subsequent calculations for enzyme quantification. Moreover, once the two chains were determined to work independently and were completely separated from each other in the predicted Bg10 and in the sequence template 1GNX PDB code [4], two catalytic sites were estimated for each protein; thus, the value of ET (that is ‘the concentration of enzyme catalytic sites’, expressed in μM) was used to estimate the k_cat_ with GraphPad Prism (version 5) software, which was calculated for one chain (58.33 kDa) and corresponded to ‘ET = 0.06172 μM’ for the 108 ng in a 30-μl reaction.

### Bg10 exhibits neutral pH, mesophilic temperature and fucose preference

**Fig 7:**
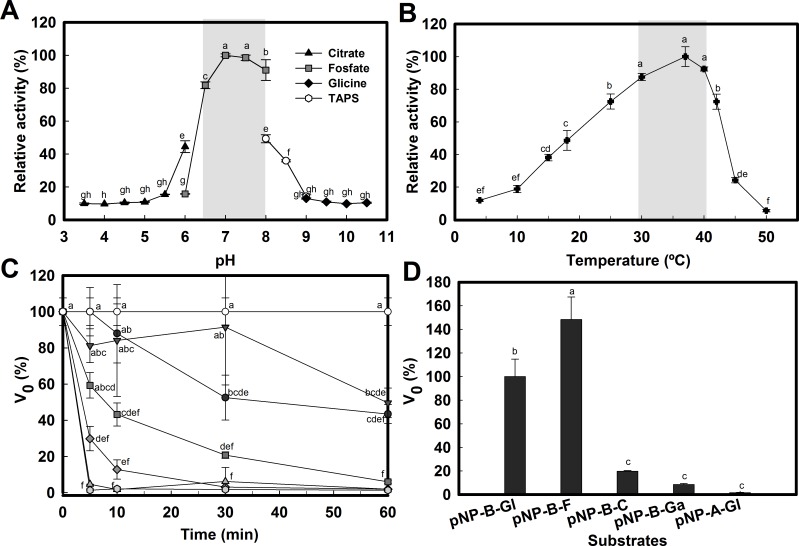
Effects of pH, temperature and different substrates on Bg10 activity. **A:** Effect of pH on β-glucosidase activity. The reactions were performed at 37°C and at pH values of 3.5 to 10.5 using 1 mM of the *p*NP-ß-D-glucopyranoside as the substrate. The reagents labelled citrate and phosphate are the sodium salts. **B:** Effect of temperature on the activity. Enzyme activity was assayed at various temperatures from 4 to 50°C in 20 mM sodium phosphate buffer (pH 7.0). **C:** Stability of β-glucosidase at the different temperatures from 10°C (control) to 50°C in 20 mM sodium phosphate buffer (pH 7.0) using 1 mM of the *p*NP-ß-D-glucopyranoside as the substrate. **D:** Enzymatic assay for Bg10 for different substrates. Enzyme activity was assayed at 37°C in 20 mM sodium phosphate buffer (pH 7.0) using 2 mM of the respective substrates: *p*NP-ß-D-glucopyranoside (*p*NP-B-Gl, which was used as the standard substrate for the activity assay), *p*NP-ß-D-fucopyranoside (*p*NP-B-F), *p*NP-ß-D-cellobioside (pNP-B-C), *p*NP-ß-D-galactopyranoside (*p*NP-B-Ga) and *p*NP-α-D-glucopyranoside (*p*NP-A-Gl). The relative activity levels or V_0_ values represent the averages of the means ± SD of triplicate reactions. Small letters in the graphics indicate the significant difference between each condition performed in the experiment, according to ANOVA and Tukey’s test at 5% probability.

The optimal conditions of reaction for Bg10 were obtained by pH, temperature, and substrate preference assays (Fig 7). The optimal pH for Bg10 was 7.0 (Fig 7A); in addition, the enzyme retained more than 80% activity within a pH range from 6.5 to 8.0, and the spectrum of utilization increased for this enzyme from slightly acidic (pH 6.5) to slightly basic conditions (pH 8) with acceptable rates of activity.

Regarding the effects of temperature on enzyme activity (Fig 7B) and stability (Fig 7C), the optimal temperature for Bg10 activity was 37°C, and substantial enzyme activity was detected in the range of 30°C to 40°C (Fig 7B), when the minimum cut-off for activity was 80%. The influence of temperature on enzyme stability (Fig 7C) revealed a high dependence of enzyme conservation to amenable temperature, as the activity decreased quickly with increasing temperature in the absence of any stabilizer, indicating that the optimum condition for enzyme storage was 10°C (Control). This sensitivity to temperature could be useful to control the period of enzyme action, allowing a process to be stopped without the final product of interest being damaged by high temperatures, a procedure applicable to the food industry. The kinetic parameters for Bg10 were calculated on basis of the liberation of *p*NP from the substrate used as the standard for the ß–glucosidase enzyme reaction—*p*NP-ß-D-glucopyranoside—under the optimum conditions of pH and temperature determined herein, and the enzyme Bg10 had the kinetic parameters V_max_, 10.81 ± 0.43 μM min^-1^; k_m_, 0.49 ± 0.12 mM; K_cat_, 175.1 ± 6.91 min^-1^, and K_cat_/K_m_, 5.91 s^-1^ mM^-1^.

To investigate the substrate preference for Bg10, the activity of purified Bg10 enzyme was tested on 2 mM of the respective chromogenic substrates (Fig 7D): *p*NP-ß-D-glucopyranoside (which was used as the standard substrate for activity assay), *p*NP-ß-D-fucopyranoside, *p*NP-ß-D-cellobioside, *p*NP-ß-D-galactopyranoside and *p*NP-α-D-glucopyranoside.

The enzyme activity towards *p*NP-ß-D-fucopyranoside was 48.3% higher than towards *p*NP-ß-D-glucopyranoside (100%). The highest activity toward pNP-ß-D-fucopyranoside was previously described for other ß-glucosidases, including Bgl3 and Td2F2 [3,13]. According to Marana (2006), although these substrates apparently share the same enzymatic site, GH1 ß-glucosidases normally prefer a fucosidic substrate because of the stronger interaction between the reactive glutamate in subsite -1 and the hydroxyl OH_2_ in these glycosides[3]. The activity levels for Bg10 towards the galactoside and α-glucoside substrates were very small (8.5% and 1.5%), which discounted the probability that Bg10 was a β-galactosidase or α-glucosidase, respectively [1]. Other β-glucosidases from Actinobacteria related to Bg10 showed little activity towards the pNP-galactoside, such as the enzyme CfBgl1 from *Cellulomonas fimi* ATCC 484, which had 5% of the activity level toward pNP-β-galactosidase [61].

Despite cellobiose being the natural substrate for β-glucosidases, for *p*NP-cellobioside, the activity was 20% of that found for the *p*NP-ß-D-glucopyranoside, suggesting that the configuration present in the first substrate ligation was less favourable to cleavage between the β-1-4 linkage with p-nitrophenolate than that found in the *p*NP-ß-D-glucopyranoside and was likely less favourable compared to the β-1-4 linkage already found between its own cellobiosidic moiety, which exhibited a glucose-β-1-4 glucosidic arrangement. According to Dairot e co-workers, the ß-glucosidase hydrolysis towards pNP-cellobioside is more efficient due to the non-reducing end of the molecule (i.e., between β-1-4 linkage from cellobiose moiety), so the glucose is immediately released, but there is a delay for liberation of *p*NP. This “lag” period for *p*NP detection is approximately 20–30 min in the literature[62]. Similarly, the Perez-Pons group used thin-layer chromatography to observe that the glucose and the moiety “NpGlcp” (and not cellobiose) were the first products from β-glucosidase hydrolysis towards pNP-cellobioside, supporting the idea that the non-reducing end is the preferential side in this chromogenic substrate [50].

The activity towards pNP-cellobioside was also similar for CfBgl1 (21%) [61], while for Bgl3 (the most similar enzyme to Bg10) showed preference for pNP-cellobioside 6% superior to pNP-glucopyranoside.

### Bg10 exhibited high stability in the presence of many ions and reagents

Enzyme activity can be affected by various ions, which might act as cofactors or as inhibitors (binding to enzymes and changing the shape of the active site) or as agents that precipitate and affect the availability and affinity of enzyme ligands. The overall effect of those many interfering compounds (metallic ions in particular) on enzyme activity can be established by comparing the activity of an enzyme in the presence and absence (control) of the interfering compounds. To assess the effects of some salts and the corresponding ions on the activity of the metagenomic ß-glucosidase, various concentrations (0 to 5 mM) of salts were tested (Table 2). The enzyme was stable in the presence of most ions, and compared to the control (without salt additions), the results showing increased significant activity was obtained for 0.5 mM lithium sulphate (increasing by nearly 37%), whereas higher concentrations (5 mM) of salts served as inhibitors for the aluminium and iron sulphates, which showed 7.5% and 39% residual activity, respectively. Bg10 activity was strongly inhibited by any concentration of cobalt and copper salts; nevertheless, the chelating effect of EDTA at a low concentration (0.1 mM) was able to restore catalytic activity in the presence of 5 mM of these salts. This finding suggested that the inhibitory effect detected in the assays is due to interference by cations (Cu^2+^ and Co^2+^) rather than anions (SO_4_^2-^ or Cl^1-^). Furthermore, the lack of variation in activity between the controls in the presence or absence of EDTA suggests that the enzyme does not depend on a cationic cofactor that was supplied by the buffer used in the reaction (sodium phosphate) or by trace elements in the reagents used. The EDTA could negatively influence some metagenomics ß-glucosidases, as with Bgl1C, which had its activity reduced to 63% in the presence of EDTA at 2 mM [1]. Thus, the ability of Bg10 to be active in the presence of EDTA could be advantageous for industrial use under conditions where the tolerance to inhibitory ions might be an important determinant, that is, it might be sufficient to include EDTA in the enzyme buffer formulation to obtain satisfactory results under adverse conditions.

**Table 2:** Effects of various salts on enzyme activity.

Relative activity (%)					
Variable	0.1 Mm	0.5 mM	1.0 mM	2.0 mM	5.0 mM
*Control*	100±7.7	100±7.7	100±7.7	100±7.7	100±7.7
^#^Control+ EDTA	N	N	N	N	102.0±13.6
Al_2_(SO_4_)^3^	114.6±4.1	123.0±2.2	112.7±2.6	109.7±4.6	^**L**^ **7.5±1.9**
CaCl_2_	103.7±3.2	93.7±2.1	96.6±8.3	92.0±2.0	86.7±34.1
CoCl_2_	^**L**^ **62.5±1.5**	^**L**^ **0.8±0.1**	^**L**^ **0.0±0.1**	^**L**^ **0.0±0.2**	^**L**^ **0.0±1.7**
^#^CoCl_2_+ EDTA	N	N	N	N	90.0±1.8
CuSO4	^**L**^ **0.0±0.2**	^**L**^ **0.6±0.1**	^**L**^ **0.0±0.3**	^**L**^ **0.3±0.3**	^**L**^ **0.0±4.2**
^#^CuSO4+ EDTA	N	N	N	N	97.5±2.7
FeSO_4_	109.6±9.1	101.7±3.8	99.3±6.5	90.1±3.7	^**L**^ **39.1±6.0**
KCl	107.3±1.1	113.5±6.6	118.1±6.1	114.9±3.7	106.4±6.8
KI	104.7±3.1	105.9±5.0	106.3±2.6	104.4±4.0	^**L**^ **69.0±20.6**
Li_2_SO_4_	^**S**^ **129.7±4.6**	^**S**^ **136.8±3.2**	110.6±7.5	120.9±1.2	^**L**^ **69.0±25.6**
MgSO_4_	91.1±2.8	98.1±2.9	95.9±1.5	91.2±4.5	^**L**^ **66.3±6.7**
MnSO_4_	98.7±7.0	97.4±5.4	99.5±3.7	91.0±6.9	78.5±7.0
NaCl	111.5±2.1	108.5±9.1	105.0±3.2	99.5±6.9	90.4±7.1

The values were calculated based on the control treatment (without additives). All measurements were performed using the *p*NP method. Standard errors of the mean values are shown.

*Reference compound/condition set as 100% activity.

^**#**^ EDTA effect on the relative activity with the control and with the respective inhibitors. N: corresponds to concentrations that were not assayed. The data was analysed using the R software ANOVA and Tukey’s test at 5% probability, therefore averages values significantly superior and lower than the control are represented by the letters **S** and **L**, respectively.

For others reagents, the ß-mercaptoethanol (1 and 5 mM) did not interfere with the Bg10 activity, whereas the SDS was entirely inhibitory (0.1–2%). For alcohols and glucose, the activity was interestingly positive, and these results revealed particular features suggesting tolerance by Bg10; these features were therefore explored further in the following section.

### Bg10 glucose stimulation and alcohol tolerance

Because some enzyme applications require stability against some products and by-products inherent in a particular process, certain enzymatic features are extremely desirable. Frequently, cases of β-glucosidases inhibited by glucose have been reported in the literature [2,11,23–25], including, for instance, inhibition of 58% of the activity of a β-glucosidase from *Monascus purpureus* in the presence of 10 mM glucose [62]. Thus, many researchers have searched for novel enzymes tolerant to glucose or have attempted to learn more about the mechanism that leads to tolerance to implement this feature in other enzymes through engineering [2,26–29]. Moreover, a demand exists for new β-glucosidases with tolerance to some alcohols present in the biofuel and beverage industry processes [12,17,63].

**Fig 8:**
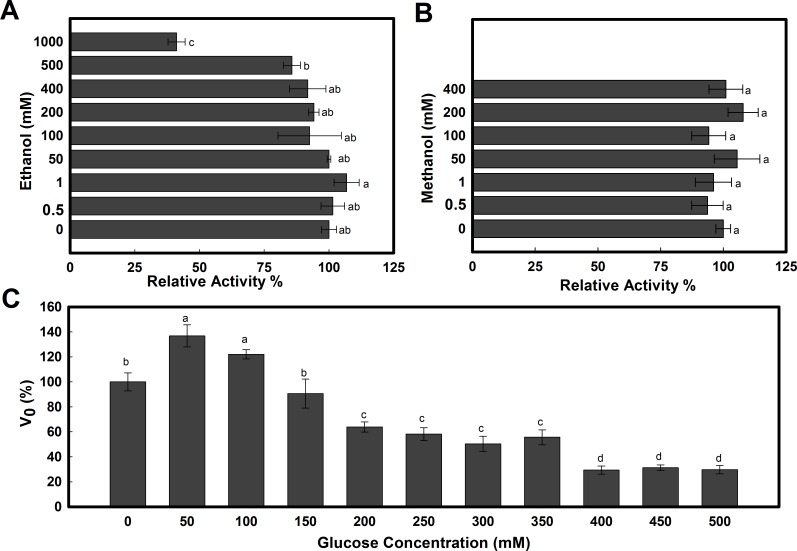
Alcohol and glucose effects on Bg10 activity. **A:** Effect of ethanol (0–1 M) on the enzyme activity. **B:** Effect of methanol (0–400 mM) on the enzyme activity. **C:** Effect of glucose tolerance on the enzyme activity. All reactions were performed in 20 mM sodium phosphate buffer (pH 7.0) at 37°C using 1 mM of *p*NP-ß-D-glucopyranoside as the substrate. The reaction mix contents with the enzyme were submitted to pre-incubation for 10 min at each treatment prior to the addition of the substrate. The relative activity or V_0_ values represent the averages of the means ± SD of triplicate reactions. Small letters in the graphics indicate the significant difference between each condition performed in the experiment, according to ANOVA and Tukey’s test at 5% probability.

Regarding these important features, an understanding of the influence of alcohols and glucose for Bg10 is also of value (Fig 8). The enzyme presented high tolerance to alcohol (Fig 8A and 8B), and small concentrations of ethanol (0.5–50 mM) and methanol (0.5–200 mM) caused little stimulation in the relative activity, exhibiting 106.8% in the presence of 1 mM ethanol and 108% in the presence of 200 mM of methanol. Daroit et al (2008) observed a similar effect of lower concentrations of ethanol on a fungal β-glucosidase (*Monascus purpureus*): a relative activity of 105–106% at low ethanol concentrations (5–50 mM) and methanol (5–250 mM) [62]. For alcohol concentrations up to 400 mM, Bg10 still presented high rates of activity for ethanol (92%) and methanol (101%) and even exhibited 41% residual activity in the presence of 1 M ethanol. In turn, the effect of low concentrations of glucose on the enzyme activity also demonstrated an enhancing effect on the Bg10 activity, which was stimulated by 36.8% and 22% with 100 and 150 mM, respectively (Fig 8C), whereas half of the activity was retained at 350 mM.

**Fig 9:**
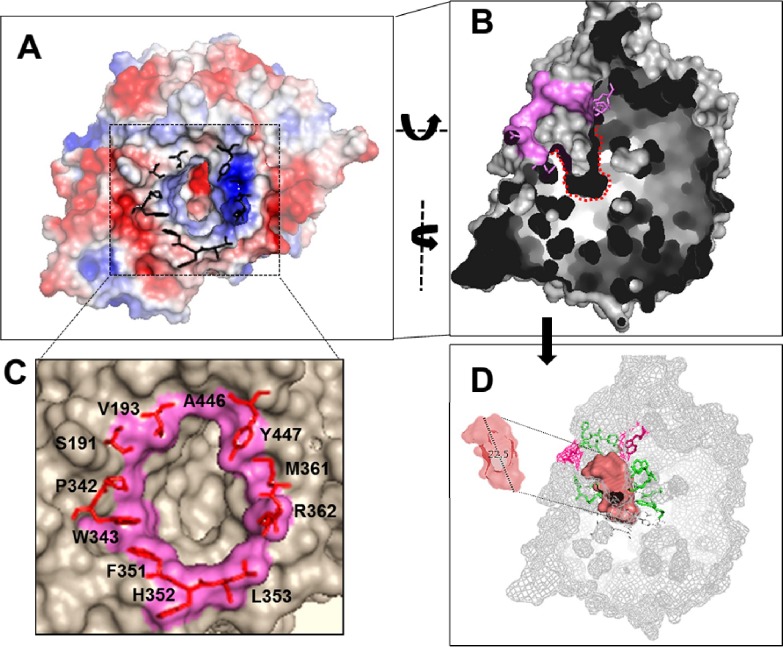
Predicted structural properties for the Bg10 enzyme (only one chain is shown). **A:** Electrostatic charge for Bg10, which was calculated using the software ‘Open-source PyMOL version 1.3’. The dotted square shows the enzyme pocket entrance, and the residue gatekeepers are shown in black. Neutral, negative and positive electrostatic regions are presented in white, red and blue, respectively. **B:** Cavity pocket cross-section showing the active site cavity marked with a dotted red line. The pink residues correspond to the entrance cavity residues exposed at this view. **C**: Gatekeeper residues located at the pocket entrance for the Bg10 enzyme. **D:** Cavity pocket cross-section (the same view shown in **B**) with details at the cavity indicating certain residues (pink, entrance residues; green, residues that delimit the pocket at its profundity, building the intern cavity). The cavity was detected using the KVFinder plugin in the software ‘Open-source PyMOL version 1.3’ with the following parameters: step size of 0.7 Å; probe in size of 0.7 Å; probe out size of 10 Å, and molecular surface to whole protein. For these parameters, the cavity had 497.664 Å^3^ of volume.

The mechanisms of the β-glucosidases product-interaction were recently attributed to the accessibility of the active site to glucose and the binding energy of this molecule at the different sites on the protein [2,31]. Among the particular features for each protein, the shape and electrostatic properties of the entrance on the active site were indicated as factors that determine the restrictions for competitiveness by glucose [31]. Using protein structure comparisons among three enzymes, the authors indicate that a more electronegative entrance charge relative to the enzyme´s charge makes the enzymes more susceptible to glucose inhibition (TrBgl2), while neutral and more positive active site entrances (HiBG and G1NkBG) are associated with glucose tolerance [31]. Taking this knowledge into account, it was investigated whether these features could be identified in the Bg10 protein prediction model; thus, the electrostatic energy of the Bg10 protein was calculated using the PyMOL tool, and the result is shown in Fig 9A. Additionally, the active pocket for Bg10 can be visualized in Fig 9B, where only one chain is presented; the cross-section shows the entrance and the depth of the chamber where the hydrolysis reaction occurs. The gatekeeper residues, in turn, are shown in Fig 9C, where they are positioned in the same view used to illustrate the electrostatic energy plot in Fig 9A, focusing on the location where the role of the residues in support of a particular electrostatic ambience at the enzyme entrance in the interference with glucose affinity is most evident [31]. Similarly, Fig 9D depicts the same view shown in Fig 9B, providing evidence of the shape and importance of the active pocket present in Bg10, according to the prediction generated by the KVFinder plugin installed in the PyMOL software. Thus, these results for Bg10 agree with the evidence provided by Giuseppe and co-workers [31], which indicates that this enzyme is also tolerant/stimulated by glucose, and its gatekeeper residues have a remarkably positive formal charge (Fig 9A and 9C). The low accessibility of the glucose to the active site due to the deep pocket [31] also seems to account for the Bg10 tolerance, which can be presumed by the presence of a deep pocket (22.5 Å), as predicted for the metagenomic β-glucosidase (Fig 9B and 9D).

### Bg10 enzyme performance against natural substrates

**Fig 10:**
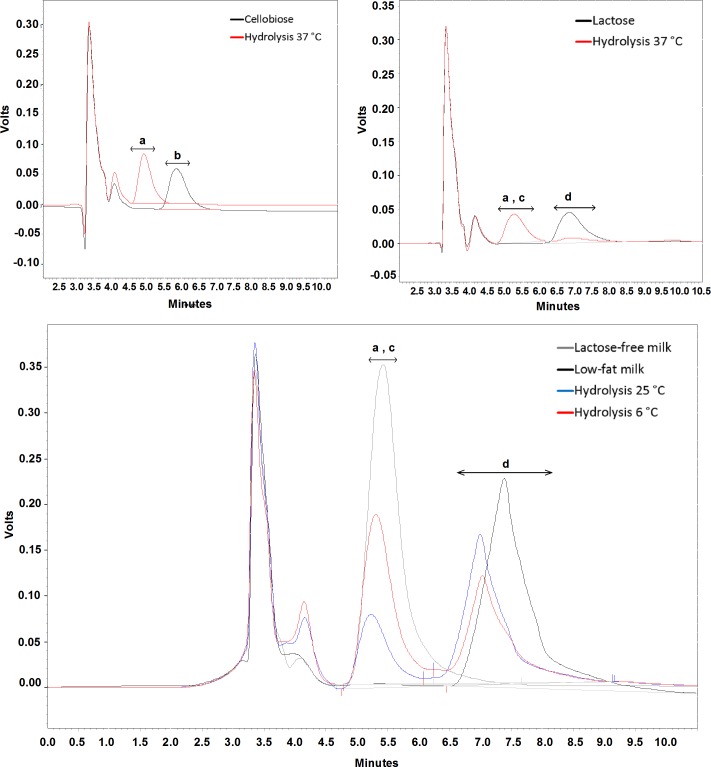
Bg10 activity on natural substrates. Cellobiose (**A**) and lactose (**B**) with (red line) and without (black line) enzyme hydrolysis at 37°C for 1 hour. **C**: Low-fat milk, hydrolysed at 25°C for 1 hour (blue line) and 6°C for 15 hours (red line), compared with the standards of low-fat milk without hydrolysis (black line) and lactose-free milk (grey line). Small letters indicate the respective sugars: **a**, glucose; **b**, cellobiose; **c**, galactose; **d**, lactose.

The enzyme activities for natural substrates were valued by HPLC for cellobiose, lactose, and milk (Fig 10). At an optimal enzyme temperature (37°C), Bg10 was able to hydrolyse the entire content of both cellobiose and lactose (Fig 10A and 10B). For milk, the Bg10 optimal temperature was avoided, as high temperatures for long periods of time might damage other natural organoleptic features in the sample, which was not the aim of the assay. Thus, the samples were submitted to room temperature (25°C) for 1 hour or to refrigeration temperature (6°C) for 15 hours. The last period is usual for the galactase digestion of lactose in ‘zero lactose’ milk process [19]. The reaction was performed without pH correction or additions other than the 120 μL of a purified enzyme preparation (100 μg of Bg10 in Tris-HCl 20 mM, NaCl 200 mM, glycerol 5%, pH 7 buffer) for each sample with 1 mL volume. After enzyme digestion, the lactose content was determined by ethanol extraction and HPLC chromatography. As shown in Fig 10C and S2 Table, the lactose contents were reduced to 45% (6°C, 15 hours) and 58% (25°C, 1 hour). Interestingly, the best result was obtained at the first temperature, at which Bg10 showed only 10% of the optimal activity (Fig 7A). These results indicate that this enzyme might be investigated as lactase for lactose-free milk production through further optimization of temperature, incubation periods, and buffers suitable for foods. Moreover, the enzyme optimum temperature, which resulted in complete hydrolysis for purified lactose, might be recommended for this process, if further assays indicate that the inclusion of this step does not affect any part of the dairy production chain. Furthermore, the complete cellobiose hydrolysis, combined with glucose and alcohol tolerances, indicated that this enzyme has great potential for applications in cellulose conversion for biofuel production.

## Conclusions

A metagenomic approach revealed a great opportunity for accessing new enzymes; therefore, Bg10 exhibits a strong potential for industrial applications. The Bg10 retained its activity under considerable concentrations of alcohols, and the effects of stimulation and high tolerance to glucose expand the enzyme’s potential for hydrolytic efficiency. The capacity to preview the enzyme shape through homology modelling highlights the structural proprieties that might support an understanding of this kinetic feature. Moreover, this enzyme is effective for use in producing milk with reduced levels of lactose, which is of interest to the food industry as an unusual application for ß-glucosidases and suggests new frontiers for biotechnology studies.

## Supporting Information

S1 Fig “Complete view”: Phylogenetic relationship between Bg10 and other previously characterized GH1 β-glucosidases. The tree was constructed using the Bayesian model with algorithmic tests to determine the better amino acid substitution matrix (Phangorn at “R” software) and phylogenetic model (Mr Bayes software) using two billion generations (Ngen) to find the better method. The scale bar indicates the number of amino acid substitutions per site. Colour code: Firmicutes, dark green; Proteobacteria, pink; Thermotogales, orange; Dictyglomales, light blue; Thermales, dark blue; “Novel green non sulphur bacteria” (NGS), light green; Petrotogales, brow; unculturable, purple; Thermobaculum, grey; Deinococcales, black.
(DOCX)

S1 Table Best hits for metagenomic ß-glucosidase Bg10 from different databases. The closest Bg10 sequences from several databases are show in this table. The data include sequences from as-yet uncharacterized enzymes described as similar as shown in the source databases.
(DOCX)

S2 Table Zero-Lactose assay. Bg10-activity toward milk samples analyzed by HPLC (High Performance Liquid Chromatography); Small letters indicate the significant difference between each condition performed in the experiment, according to ANOVA and Tukey’s test at 5% probability.
(DOCX)
