# Supplementary material for: Bg10: A Novel Metagenomics Alcohol-Tolerant and Glucose-Stimulated GH1 ß-Glucosidase Suitable for Lactose-Free Milk Preparation
Source: PLoS One. 2016 Dec 21;11(12):e0167932. doi: 10.1371/journal.pone.0167932 (PMC5176175; doi:10.1371/journal.pone.0167932)
Supplement: S1 Fig — The tree was constructed using the Bayesian model with algorithmic tests to determine the better amino acid substitution matrix (Phangorn at “R” software) and phylogenetic model (Mr Bayes software) using two billion generations (Ngen) to find the better method. The scale bar indicates the number of amino acid substitutions per site. Colour code: Firmicutes, dark green; Proteobacteria, pink; Thermotogales, orange; Dictyglomales, light blue; Thermales, dark blue; “Novel green non sulphur bacteria” (NGS), light green; Petrotogales, brow; unculturable, purple; Thermobaculum, grey; Deinococcales, black. (DOCX) [file pone.0167932.s001.docx]

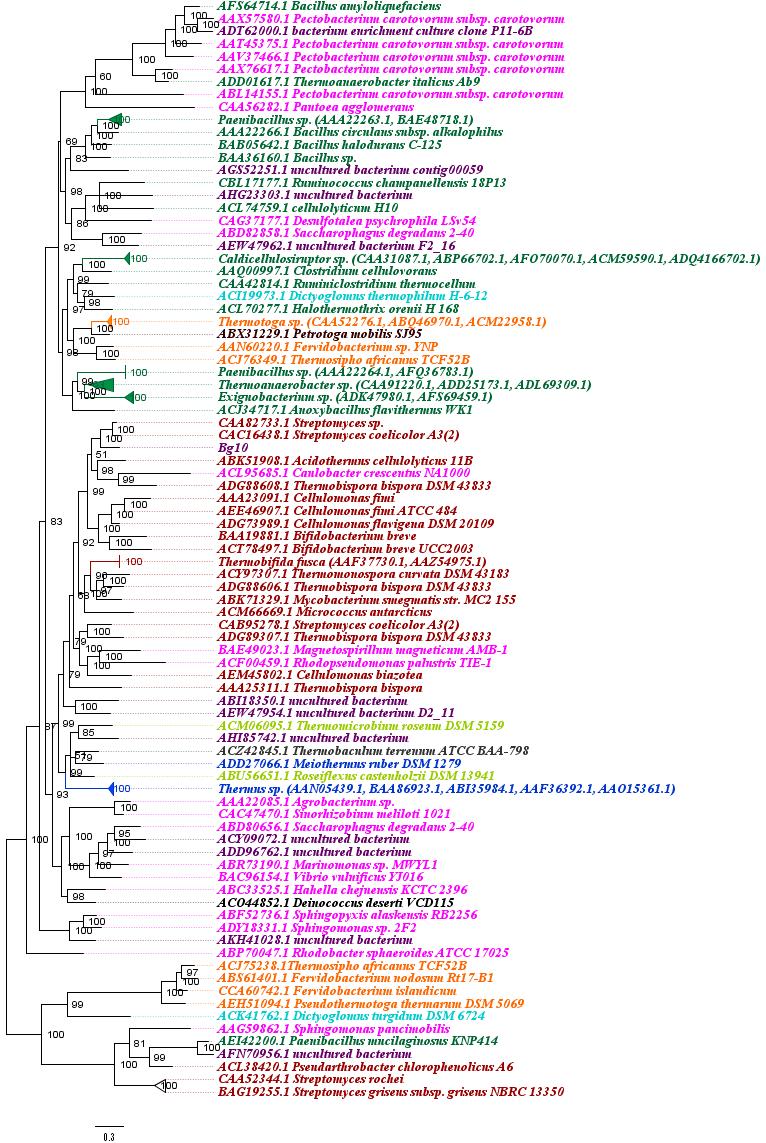


**S1 Fig.** **“Complete view”: Phylogenetic relationship between Bg10 and other previously characterized GH1 β-glucosidases.** The tree was constructed using the Bayesian model with algorithmic tests to determine the better amino acid substitution matrix (Phangorn at “R” software) and phylogenetic model (Mr Bayes software) using two billion generations (Ngen) to find the better method. The scale bar indicates the number of amino acid substitutions per site. Colour code: Firmicutes, dark green; Proteobacteria, pink; Thermotogales, orange; Dictyglomales, light blue; Thermales, dark blue; “Novel green non sulphur bacteria” (NGS), light green; Petrotogales, brow; unculturable, purple; Thermobaculum, grey; Deinococcales, black.
