# Supplementary material for: Bg10: A Novel Metagenomics Alcohol-Tolerant and Glucose-Stimulated GH1 ß-Glucosidase Suitable for Lactose-Free Milk Preparation
Source: PLoS One. 2016 Dec 21;11(12):e0167932. doi: 10.1371/journal.pone.0167932 (PMC5176175; doi:10.1371/journal.pone.0167932)
Supplement: S1 Table — The closest Bg10 sequences from several databases are show in this table. The data include sequences from as-yet uncharacterized enzymes described as similar as shown in the source databases. (DOCX) [file pone.0167932.s002.docx]

**S1 Table -** **Best hits for metagenomic ß-glucosidase Bg10 from different databases**.

The closest Bg10 sequences from some databases are show in this table. The data include sequences from no characterized enzymes descripted as same as showed in source databases.

| **Web sever** | **Enzyme/organism** | **% Identity** | **Accession*** |
| --- | --- | --- | --- |
| BLASTp, Non- redundant Database | ß-galactosidase (*Streptomyces* sp.) | 88% | WP_033177502.1 |
|  | ß-glucosidase (*Streptomyces* sp.) | 84% | WP_031523150.1 |
|  | ß-galactosidase (*S. yeochonensis*) | 80% | WP_037907004.1 |
| BLASTP, Metagenome Database | hypothetical protein LCGC14 1888300 (marine metagenome) | 48% | KKL92078.1 |
|  | unnamed protein product [marine metagenome] | 42% | GAG70566.1 |
|  | GH1 Beta-glucosidase Td2f2 (Soil Metagenome) | 48% | 3WH5_A |
| BLASTp, Protein Database | ß-glucosidase (*Streptomyces* sp*. QM-B814*) | 69% | 1GNX_A^1^ |
|  | ß-glucosidase (uncultured bacterium) | 49% | 4HZ6_A^2^ |
|  | ß-glucosidase (Soil metagenome) | 49% | 3CMJ_A^3^ |
|  | ß-glucosidase (*Micrococcus antarcticu*s) | 49% | 3W53_A^4^ |
| Swiss Model | ß-glucosidase (*Streptomyces* sp*.*) | 69.83% | 1gnx.1A^1^ |
|  | ß-glucosidase (*Micrococcus antarcticu*s) | 51.32% | 3w53.1.A^4^ |
|  | ß-glucosidase (uncultured bacterium) | 51.70% | 4hz6.1.A^2^ |
|  | ß-glucosidase (Soil metagenome) | 51.82% | 3cmj.1.A^3^ |
| Phyre2 | (Trans)glycosidases (*Streptomyces* sp*.*) | 70% | d1gnxa^1^ |
|  | (Trans)glycosidases (*Bacillus circulans* sp*. Alkalophilus*) | 47% | d1qoxa |
|  | ß-glucosidase (*Micrococcus antarcticus*) | 49% | c3w53A^4^ |
|  | ß-glucosidase (uncultured bacterium) | 51% | c3fiyA (4hz6)^2^ |

*****The numbers indicate the same protein identified in a different database
