# Supplementary material for: Bg10: A Novel Metagenomics Alcohol-Tolerant and Glucose-Stimulated GH1 ß-Glucosidase Suitable for Lactose-Free Milk Preparation
Source: PLoS One. 2016 Dec 21;11(12):e0167932. doi: 10.1371/journal.pone.0167932 (PMC5176175; doi:10.1371/journal.pone.0167932)
Supplement: S2 Table — Bg10-activity toward milk samples analyzed by HPLC (High Performance Liquid Chromatography); Small letters indicate the significant difference between each condition performed in the experiment, according to ANOVA and Tukey’s test at 5% probability. (DOCX) [file pone.0167932.s003.docx]

S2 Table - **Zero-Lactose assay.** Bg10-activity toward milk samples analysed by HPLC (High Performance Liquid Chromatography)

| Samples | Lactose^#^ (%) | Time (hour) | Temp (°C) |
| --- | --- | --- | --- |
| Lactose-free milk | 0.00±0.00^d^ | 0 | 6 |
| Low-fat milk (LFM) | 99.97±0.06^a^ | 0 | 6 |
| Hydrolyzed-LFM (room temperature) | 58.63±0.25^b^ | 1 | 25 |
| Hydrolyzed-LFM (refrigerated) | 44.96±0.06^c^ | 15 | 6 |

^#^Small letters indicate the significant difference between each condition performed in the experiment, according to ANOVA and Tukey’s test at 5% probability
